# Supplementary material for: Predicting Climate Change Impact on the Habitat Suitability of the Schistosoma Intermediate Host Oncomelania hupensis in the Yangtze River Economic Belt of China
Source: Biology (Basel). 2024 Jun 27;13(7):480. doi: 10.3390/biology13070480 (PMC11273679; doi:10.3390/biology13070480)
Supplement: Supplementary file 1 [file biology-13-00480-s001.zip › supplementary materials.pdf]

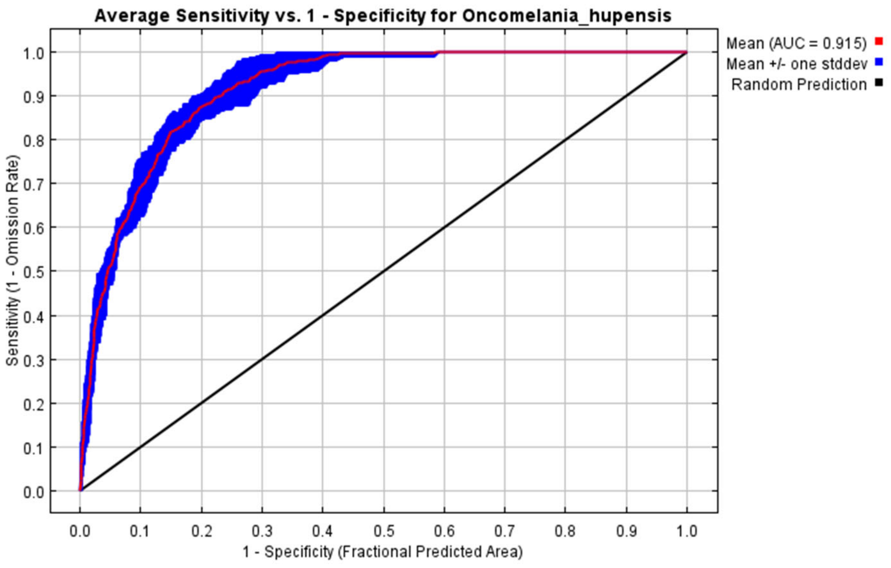

Figure S1. Reliability test of the distribution model created for *Oncomelania hupensis*. The receiver operating characteristic (ROC) curve and average area under curve (AUC) values for the optimized model over 10 replicate runs were shown in red, while blue margins show ± standard deviation (SD) calculated for 10 replicates.

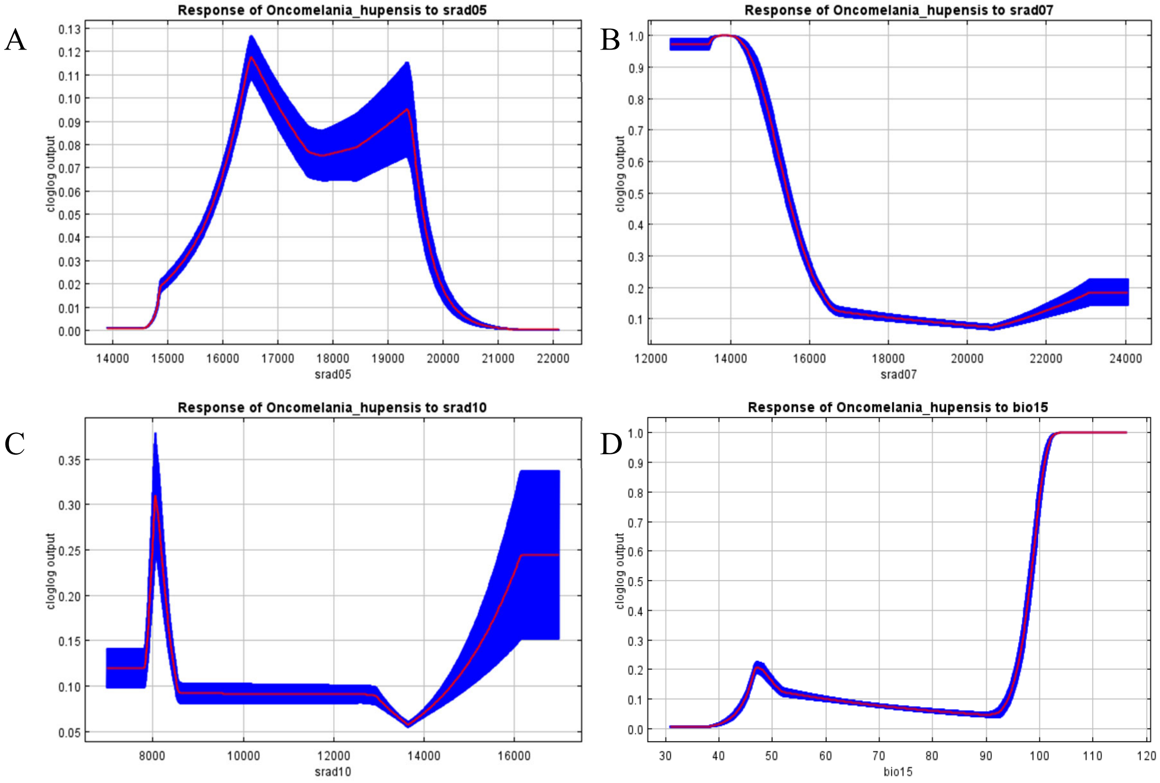

Figure S2. Response curves of MaxEnt models for environmental variables.The red curves represent average value over 10 replicate runs, while blue margins represented ± SD calculated for 10 replicates: (**A**) srad05=Solar radiation in May, (**B**) srad07=Solar radiation in July, (**C**) srad10=Solar radiation in October, (**D**) bio15=Precipitation Seasonality.

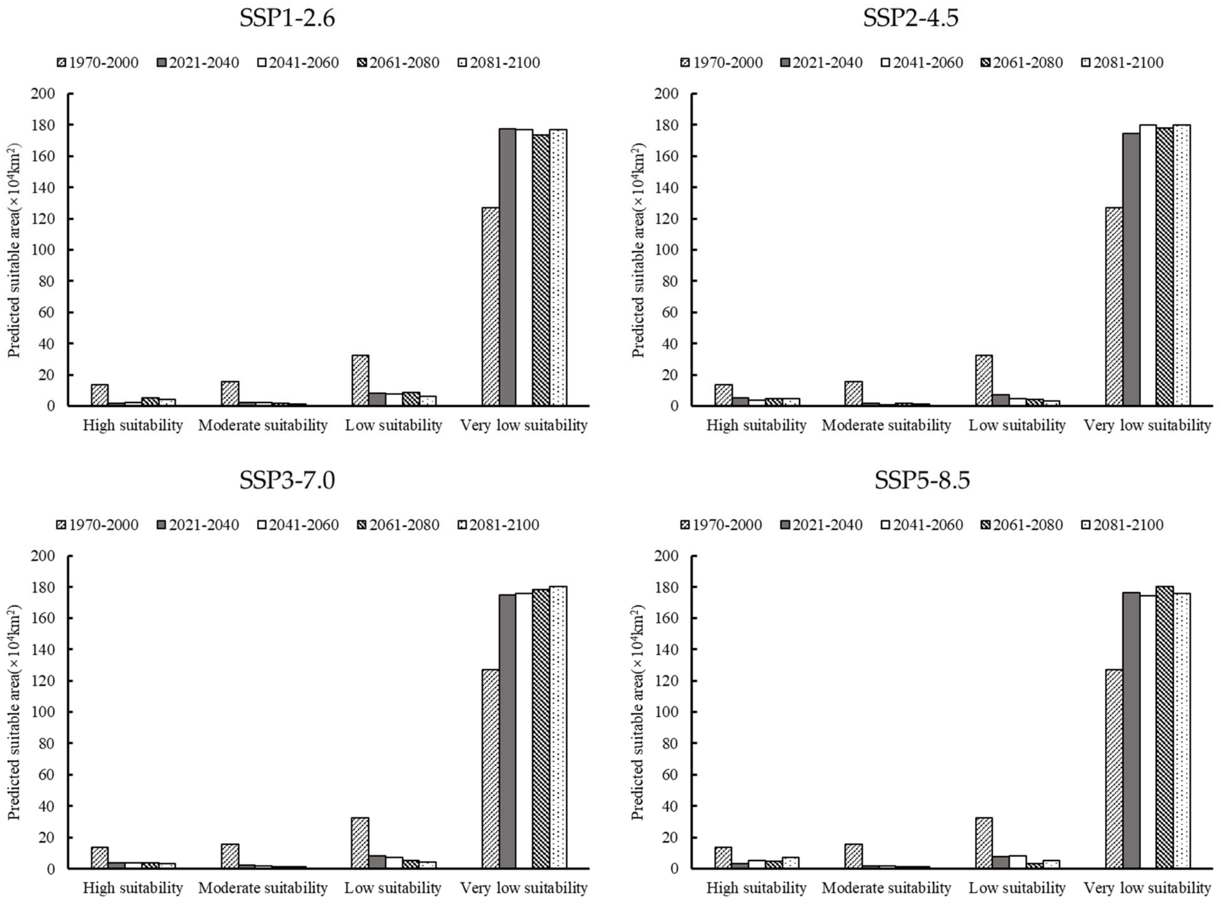

Figure S3. Suitable habitat area of *Oncomelania hupensis* in the Yangtze River Economic belt in China under current and future climate scenarios.
